# Atomic-Scale Tuning of the Charge Distribution by Strain Engineering in Oxide Heterostructures

**DOI:** 10.1021/acsnano.1c05220

**Published:** 2021-09-30

**Authors:** Yu-Mi Wu, Y. Eren Suyolcu, Gideok Kim, Georg Christiani, Yi Wang, Bernhard Keimer, Gennady Logvenov, Peter A. van Aken

**Affiliations:** † Max Planck Institute for Solid State Research, Heisenbergstrasse 1, 70569 Stuttgart, Germany; ‡ Department of Materials Science and Engineering, Cornell University, Ithaca, New York 14853, United States

**Keywords:** thin film, heterostructure, strain, charge transfer, scanning transmission electron microscopy, electron energy-loss spectroscopy, molecular beam epitaxy

## Abstract

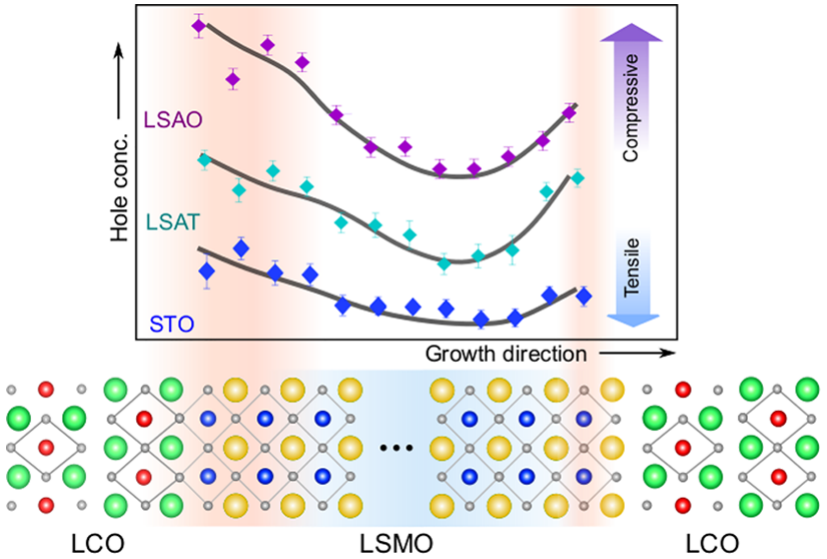

Strain engineering of complex oxide heterostructures has provided routes to explore the influence of the local perturbations to the physical properties of the material. Due to the challenge of disentangling intrinsic and extrinsic effects at oxide interfaces, the combined effects of epitaxial strain and charge transfer mechanisms have been rarely studied. Here, we reveal the local charge distribution in manganite slabs by means of high-resolution electron microscopy and spectroscopy via investigating how the strain locally alters the electronic and magnetic properties of La_0.5_Sr_0.5_MnO_3_–La_2_CuO_4_ heterostructures. The charge rearrangement results in two different magnetic phases: an interfacial ferromagnetically reduced layer and an enhanced ferromagnetic metallic region away from the interfaces. Further, the magnitude of the charge redistribution can be controlled via epitaxial strain, which further influences the macroscopic physical properties in a way opposed to strain effects reported on single-phase films. Our work highlights the important role played by epitaxial strain in determining the spatial distribution of microscopic charge and spin interactions in manganites and provides a different perspective for engineering interface properties.

In complex oxide heterostructures, a controlled modification of the charge-carrier density at the interface can yield a wide variety of phenomena that are absent in bulk materials.^1−3^ Many studies in this field have focused on the coupling between manganites and cuprates.^4−7^ It has been predicted that charge transfer from a manganite to a cuprate occurs because of the difference between their chemical potentials.^8^ X-ray spectroscopy studies of La_2/3_Ca_1/3_MnO_3_/YBa_2_Cu_3_O_7_ interfaces have indeed demonstrated a charge transfer of ∼0.2 *e*^–^ per Cu ion from Mn to Cu, causing a change in orbital occupation and an induced net magnetic moment in the cuprate.^9^ In addition, the spatial evolution of the electronic ground state at the interface has been also observed.^10,11^ The length scale of the charge transfer, measured by scanning tunneling microscopy, was suggested to be in the subnanometer range,^12^ and the spatial broadening of the electronic transition is correlated with the rougher interface. Meanwhile, electron energy-loss spectroscopy measurements revealed an electron enrichment in the manganite layer with a few nanometer thickness near the interface as a result of orbital hybridization and Cu/Mn substitution.^13,14^ These observations suggest that disorder effects are an important factor in attempts to understand the spatial correlations in such systems and to obtain precise control of the electronic structure at the interface.

Strain can provide an additional handle to manipulate the interfacial coupling between two materials. An anisotropic hopping between orbitals can be induced by structural changes and cause an orbital ordering.^15−18^ In single-layer manganite thin films, the elongation or compression of MnO_6_ octahedra can split the degenerate e_g_ levels, lowering either the 3*z*^2^–*r*^2^ or the *x*^2^–*y*^2^ state based on the Jahn–Teller effect.^19^ Experimentally, the magnetic ground state of La_0.5_Sr_0.5_MnO_3_ (LSMO) is observed to change from an insulating and antiferromagnetic (AF) *C*-type, to a metallic and ferromagnetic (FM), and finally to an in-plane conducting and AF *A*-type phase by changing the tetragonality, *c*/*a* ratio, from 1.04 (compressive strain) to 0.98 (tensile strain).^20−23^ Thus, by varying the strain condition the preferential orbital occupation changes, one can directly modify the electronic and magnetic properties of the material. However, the role of the epitaxial strain for the charge transfer at the interface as well as the interfacial magnetic coupling in cuprate/manganite heterostructures is not yet well understood and explored. A comprehensive picture of the interplay between the lattice degrees of freedom and the electronic structure still calls for a detailed investigation with atomic accuracy.

Here, we provide a systematic nanoscopic investigation of strain and interface effects in La_0.5_Sr_0.5_MnO_3_ (LSMO) layer inserted between insulating antiferromagnetic La_2_CuO_4_ (LCO) layers grown on three different substrates (LCO/LSMO/LCO-substrate system) with different lattice spacings. Using scanning transmission electron microscopy (STEM) combined with electron energy-loss spectroscopy (EELS), the detailed chemical composition and the changes of the local Mn valence in the system can be probed at the atomic scale near the interfaces. An asymmetric charge distribution near interfaces within the manganite layers is observed: Hole accumulation near interfaces suppresses the magnetization, giving rise to an exchange-bias effect. Away from the interfaces, the ferromagnetic order is recovered by an electron enrichment. Different from strain effects reported on single-phase films, we find that the charge redistribution in manganite layers is correlated with the interfacial Cu/Mn intermixing as well as the substrate-induced strain, which in turn alters the charge transfer at the interface and the physical properties of the LCO/LSMO/LCO-substrate system.

## Results and Discussion

### Structural Characterization

LCO/LSMO/LCO trilayers with 10-unit-cell thick LSMO and 4-unit-cell thick top and bottom LCO layers were grown on (100) SrTiO_3_ (STO), (100) (LaAlO_3_)_0.3_-(Sr_2_AlTaO_6_)_0.7_ (LSAT), and (001) LaSrAlO_4_ (LSAO) single-crystalline substrates by ozone-assisted molecular beam epitaxy (MBE).^24^ We choose the La_1–*x*_Sr_*x*_MnO_3_ with *x* = 0.5 compound with a thickness of 10 unit cells (∼4 nm), as its physical properties are highly sensitive to the interfacial perturbations and are close to the critical value of the dead layer effect in manganites.^25−27^ The in-plane lattice parameters for STO, LSAT, and LSAO substrates are 3.905, 3.87, and 3.75 Å, respectively. LSMO with the pseudocubic lattice parameter, *a*_0_, of 3.86 Å is under tensile and compressive strain on STO and LSAO, respectively, and a negligible strain on LSAT. The lattice mismatch values referred to LSMO bulk compounds, δ = (*a*_0_ – *a*_substrate_)/*a*_0_ × 100%, are −1.2% (STO), −0.3% (LSAT), and 2.9% (LSAO). From the measured Mn–Mn interatomic distances for each layer (Figure S3), the averaged *c*/*a* ratios of the LSMO layers on LSAO, LSAT, and STO are 1.08, 1.00, and 0.98, respectively (Table S1).

**Figure 1:**
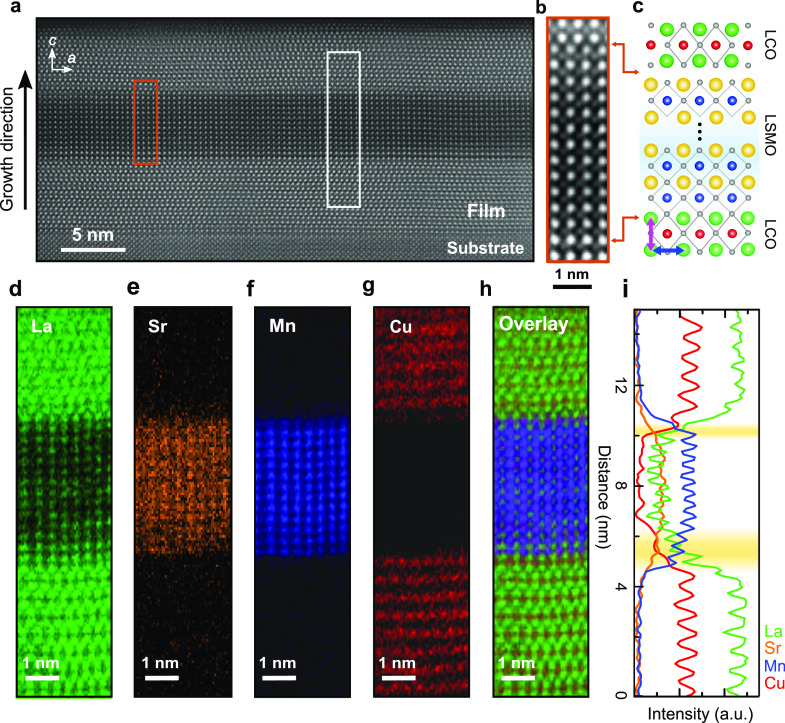
Overview of the LCO/LSMO/LCO interface lattice structure. (a) Low-magnification STEM-HAADF image of the film on LSAT(001). (b) High magnification of the area highlighted by the orange rectangle in (a). (c) Schematic arrangement of atoms showing two different stacking sequences at two interfaces, indicated by orange arrows. The blue and magenta arrows indicate the in-plane and out-of-plane directions, respectively. (d–g) Elemental concentration maps of La *M*_4,5_, Sr *L*_2,3_, Mn *L*_2,3_, and Cu *L*_2,3_ edges, respectively, from the area of the white rectangle in (a). (h) Overlay map with La in green, Sr in orange, Mn in blue, and Cu in red. (i) Horizontally integrated intensity profiles of La (green), Sr (orange), Mn (blue), and Cu (red) distributions obtained from the maps. The maps in grayscale and intensity profiles for whole compositions are shown in Figure S4. The nominal interface is where the Sr concentration reaches 50% on the A-site sublattice. The yellow shaded regions indicate the width of cation intermixing at both interfaces, determined by the region from the onset of the Sr intensity profile to the nominal interface.

To confirm the structural quality of the films, we first investigate the LCO/LSMO/LCO trilayer on the LSAT (001) substrate as a representative sample. The low-magnification STEM high-angle annular dark-field (HAADF) image (Figure 1a) demonstrates a good macroscopic crystal quality with structurally coherent LCO/LSMO and LSMO/LCO interfaces. Similar to prior work,^28,29^ we observe differences in both the Mn distribution and the atomic stacking sequences at the top and bottom interfaces (Figure 1b,c). A direct Cu–O–Mn bonding at the bottom interface is followed by an indirect contact at the top interface. In order to explore the elemental distribution, we acquire atomic-resolution 2D elemental maps across the two interfaces. Figure 1d–g displays La (green), Sr (orange), Mn (blue), and Cu (red) maps, respectively. The superimposed overlay (Figure 1h) and the normalized intensity profiles (Figure 1i) of each element show that the bottom LCO/LSMO interface has a stronger intermixing that spreads over ∼1.5 nm, while the top LSMO–LCO interface is abrupt. Away from the interfaces, the La and Sr concentration remains the same. The trilayers grown on STO and LSAO show similar results (Figures S5, S6) suggesting that the asymmetric cation intermixing at these interfaces is largely independent of the magnitude of the substrate-induced strain and possibly correlates with different stacking sequences and growth kinetics instead.^24,30^

### Electronic and Magnetic Properties of Trilayers

**Figure 2:**
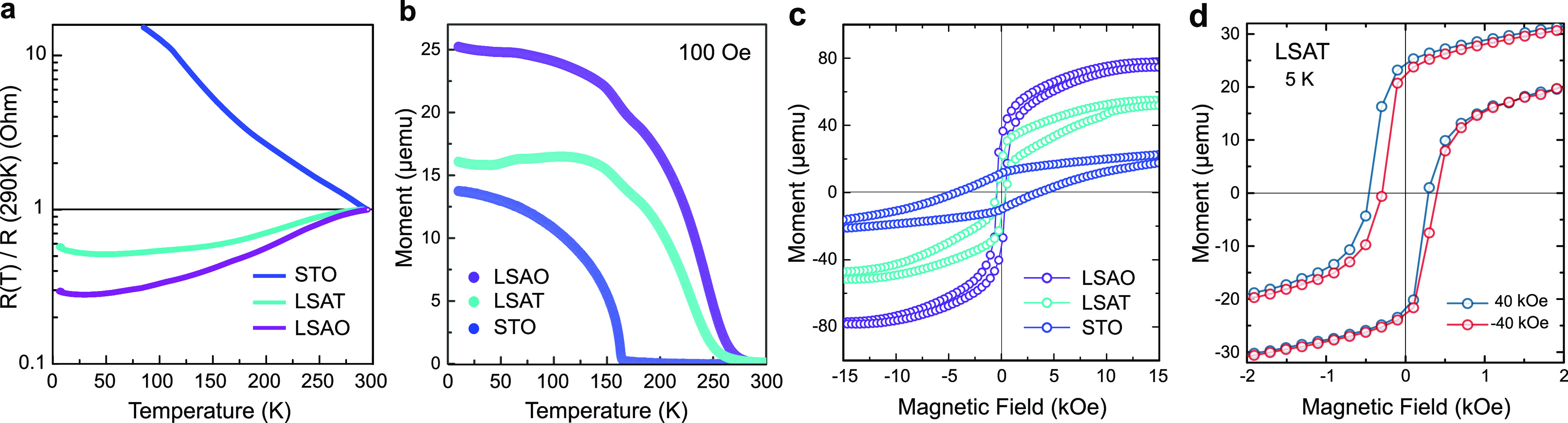
Physical properties of LCO/LSMO/LCO trilayers. (a) Normalized electrical resistance curves of films on STO, LSAT, and LSAO. (b) Comparison of temperature-dependent magnetization for all films. The curves were measured after field cooling the samples in a 100 Oe in-plane field. (c) Magnetic hysteresis loops for all films measured at 5 K. (d) Hysteresis loops at 5 K showing an exchange bias field of ∼70 Oe in the film on LSAT. Full magnetic hysteresis loops are presented in Figure S7.

Next, we turn our attention to transport and magnetic measurements of all three LCO/LSMO/LCO trilayers. The resistance vs temperature (*R–T*) curves in Figure 2a are normalized to the resistance at 290 K to reveal the differences at low temperatures. The trilayer grown on STO is more semiconductor-like with diverging resistance as *T* → 0, which agrees well with the expected semiconducting state in half-doped LSMO.^25,26^ However, films grown on LSAT and LSAO show metallic behavior. Meanwhile, noticeable changes in the magnetic interactions for the three samples are also observed (Figure 2b,c). The Curie temperature, *T*_C_, as well as the saturation magnetization increases, and accordingly, the resistivity decreases, consistent with the well-known behavior of manganites.^31^ The measured *T*_C_ for the three films are determined to be ∼163, 230, and 247 K on STO, LSAT, and LSAO, respectively. Note that the Néel temperature for the antiferromagnetic LCO cannot be determined due to its weak magnetic signal. The measured magnetism of the films is, therefore, dominated by the LSMO layer. Moreover, the trilayer structure is identical on all three substrates, so the enhanced magnetism should arise from the enhanced double-exchange contribution to the magnetic interactions. Prior studies on epitaxial LSMO thin films show that compressive epitaxial strain tends to reduce *T*_C_ and suppress the magnetization in LSMO.^20,21^ Thus, the compressive strain of the LCO/LSMO/LCO trilayer on LSAO is expected to weaken the magnetism. However, we observe that the film on LSAO shows the largest magnetic moment, while for STO the magnetization of the film is reduced with a lowered *T*_C_. This unexpected behavior suggests that the magnetic and electronic properties of the trilayers cannot be simply ascribed to the induced epitaxial strain. In addition, all samples exhibit nonzero values of the exchange bias at 5 K, consistent with previously reported values.^28^ The representative hysteresis loops of the film on LSAT clearly demonstrate the characteristic exchange-bias shift along the magnetic-field axis in Figure 2d. These results suggest that the existence of magnetic frustration near interfaces originates from an exchange coupling of the ferromagnetic layer to the antiferromagnetic interface layer.^32−35^ Detailed information about the measured exchange bias and zero-field-cooled magnetization curves for all samples can be found in the Figures S8 and S9.

### Probing Charge Variation Across the Interfaces

**Figure 3:**
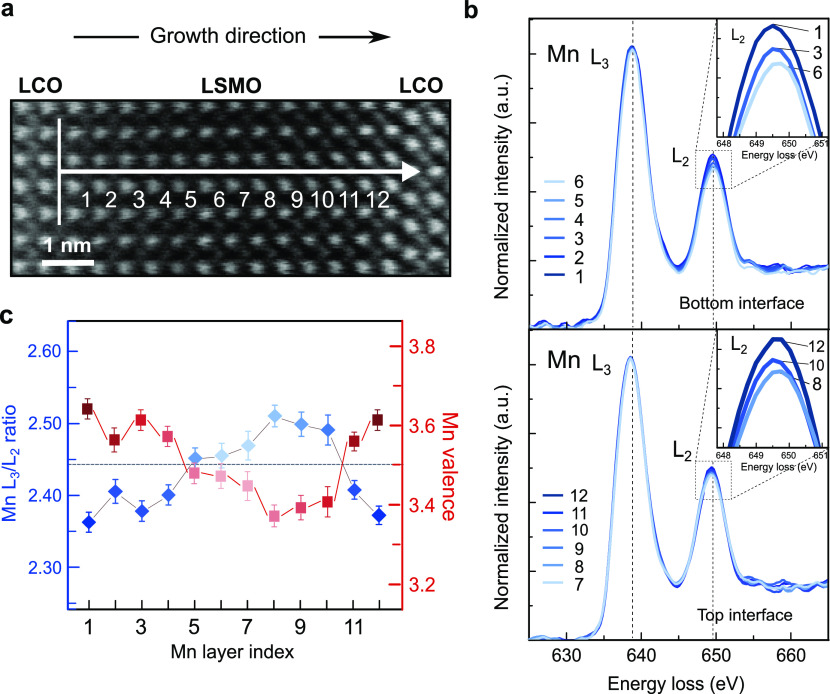
Electronic transition across the interfaces. (a) STEM-ADF image of LCO/LSMO/LCO film on LSAT. The white arrow indicates the region, where the EELS spectra were acquired. The vertical line indicates the averaging width while scanning. (b) Layer-resolved Mn *L*_2,3_ edge spectra collected from the bottom interface into central LSMO, layers 1–6 in (a) and from central LSMO to the top interface, layers 7–12 in (a). Zoomed views of the Mn *L*_2_ white-line intensity are shown in the insets. The spectra were processed through a power-law background subtraction followed by a normalization to the integrated intensity under Mn *L*_3_ white line. The Mn *L*_2_ white-line intensity increases progressively close to the interface, indicative of an increase in the Mn valence. (c) Local variation of in the Mn *L*_3_/*L*_2_ intensity ratio (blue) and the corresponding Mn valence (red) within LSMO. The dashed gray line represents the nominal 3.5+ Mn valence for stoichiometric LSMO assuming charge neutrality. The Mn *L*_3_/*L*_2_ intensity ratio was calculated using the white-line ratio method of ref (37). The standard error of integrated Mn *L*_2,3_ intensity values was used to calculate error bars of intensity ratios. Valence states and their error bars were calculated by corresponding errors of Mn *L*_3_/*L*_2_ intensity ratios and the formula in ref (37).

The exchange interaction between Mn^3+^ and Mn^4+^ ions in manganites is at root of the correlation between conductivity and ferromagnetism.^36^ Herewith, we focus on changes in the local Mn valence by probing the Mn *L*_2,3_ edge fine structures, which reflect the unoccupied local Mn 3*d* density of states.^37^ The evolution of the Mn *L*_2,3_ edge spectra on each atomic layer within LSMO of the trilayer on LSAT is shown (Figure 3). A large width perpendicular to the scanning direction was averaged along the linescan to avoid any beam damage to the film and to increase the signal-to-noise ratio of the linescan, which in turn ensures the accuracy of the valence determination. Spectra on layers 1–6 in Figure 3a were obtained from the bottom interface to the central LSMO layers, while layers 7–12 were scanned starting from the central layers to the top interface through the same scan. The Mn *L*_2,3_ spectra (Figure 3b) show a clear progressive increase of the *L*_2_ intensity from the central layers to both interfaces, as an indication of the valence changes within LSMO. To quantify this effect, the atomic-layer-resolved *L*_3_/*L*_2_ intensity ratios and corresponding valence states^37^ were determined from layers 1–12 and are presented in Figure 3c. The Mn valence profile exhibits an asymmetric shape near the two interfaces. The bottom interface displays a wide region of an increased Mn valence close to 3.6+ over a three-monolayer-broad region, while the top interface displays a more narrow region of approximately one monolayer. The spatial extent of these regions agrees well with the trend observed in the B-site intermixing at both interfaces (Figure 1i). More importantly, valence changes not only occur near interfaces but also extend to central Mn layers: Away from the interface (layers 5–10), a significantly lower valence state than the expected value 3.5+ is observed. This suggests that the underlying dopant-concentration profiles within LSMO do not play a dominant role in the changes in the Mn valence. Instead, the presence of a charge redistribution occurs in our system.

### Strain-Tuned Local Charge Redistribution

**Figure 4:**
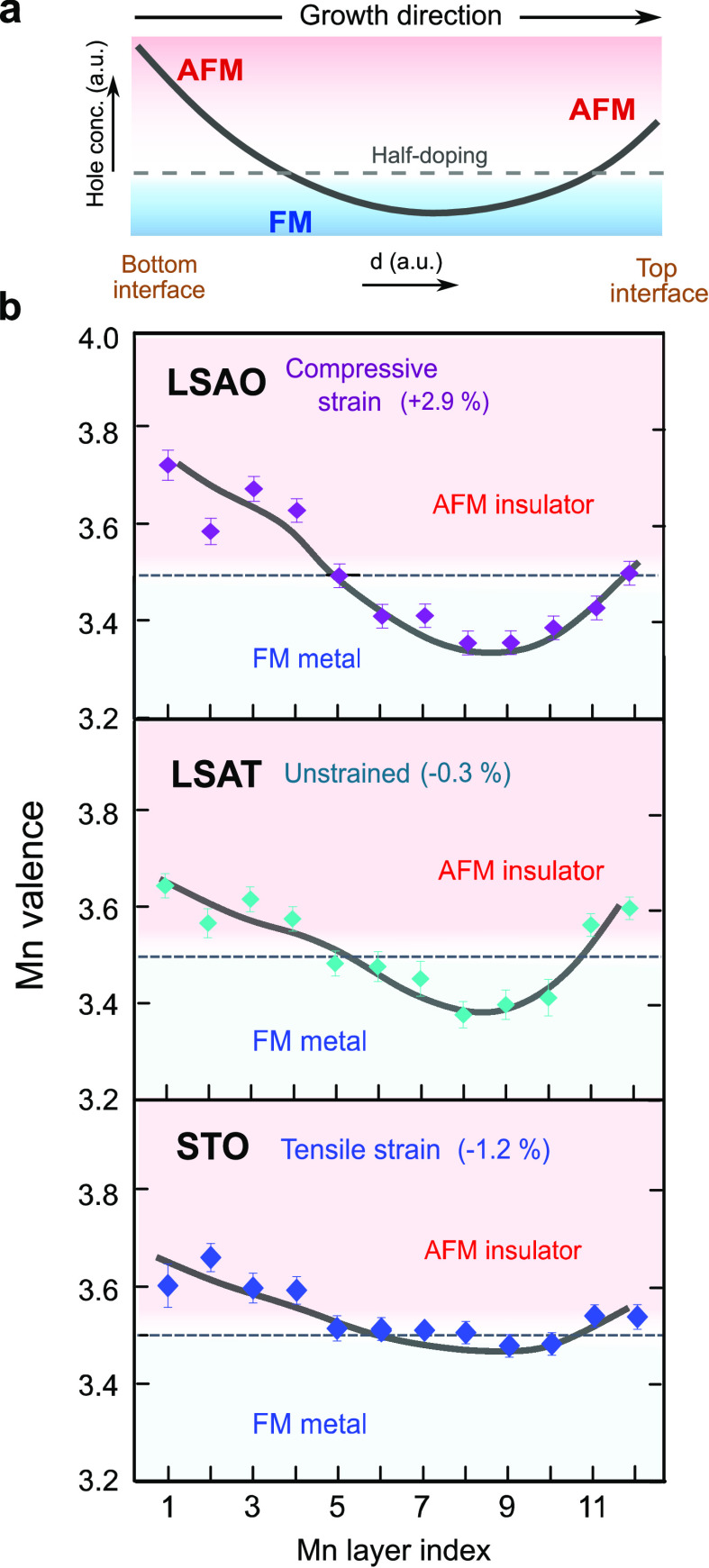
Phase mapping of relative Mn valence in manganite layers. (a) Sketch of the expected asymmetric hole profile in LSMO as a consequence of the cation intermixing at the interfaces. The dashed gray line represents the nominal hole concentration of *x* = 0.5 in LSMO. (b) Map of local Mn valence and corresponding local magnetic phase relative to bulk La_0.5_Sr_0.5_MnO_3_ in LSMO layers on LSAO, LSAT, and STO, respectively. The boundaries of associated magnetic and electronic phases are estimated from the bulk LSMO phase diagram. Thick solid lines on charge profiles are guides to the eye.

The overall trend of the observed asymmetric hole profile within LSMO layers (*cf*. Figure 3c) is depicted in Figure 4a. To explore the origin of unexpected physical properties that we observe, comprehensive analyses of Mn valence distributions are extended to all trilayers grown on the three substrates in Figure 4b. We estimate the local electronic and magnetic phase present in LSMO by comparing the measured Mn valence with the Mn doping relative to its bulk-like state. We find that for all samples a significantly increased Mn valence near the bottom interface (first to fourth Mn layer) leads to a formal local doping close to the *x* = 0.6 antiferromagnetic state. This is consistent with previous theoretical model calculations and experimental polarized neutron reflectometry studies showing the lack of carriers leading to magnetic and electronic phase separation^31,38,39^ and a reduced FM due to Mn^4+^–Mn^4+^ superexchange antiferromagnetic interaction at the cuprate/manganite interface.^14,28^ Away from the interfaces, the magnitude of electron enrichment due to the presence of a lowered Mn valence in LSMO differs significantly for the three substrates. This suggests that the magnetization and conductivity within LSMO are mainly dominated by Mn–Mn double-exchange interactions in the central Mn layers (fifth to tenth Mn layers). The magnetization as well as the conductivity increase as the electron enrichment increases within the central Mn layers. Under compressive strain on LSAO, central Mn layers are close to *x* = 0.3 for a bulk-like FM phase, which corresponds to the highest ferromagnetic moment and lowest resistivity in the phase diagram. On the other hand, in the case of the tensile strain for the STO substrate, the weakened charge delocalization leads to a reduction of the total magnetization, Curie temperature, and metallicity, compared to the other two films.

### Ca-Doping: LCO/LCMO/LCO Trilayer

**Figure 5:**
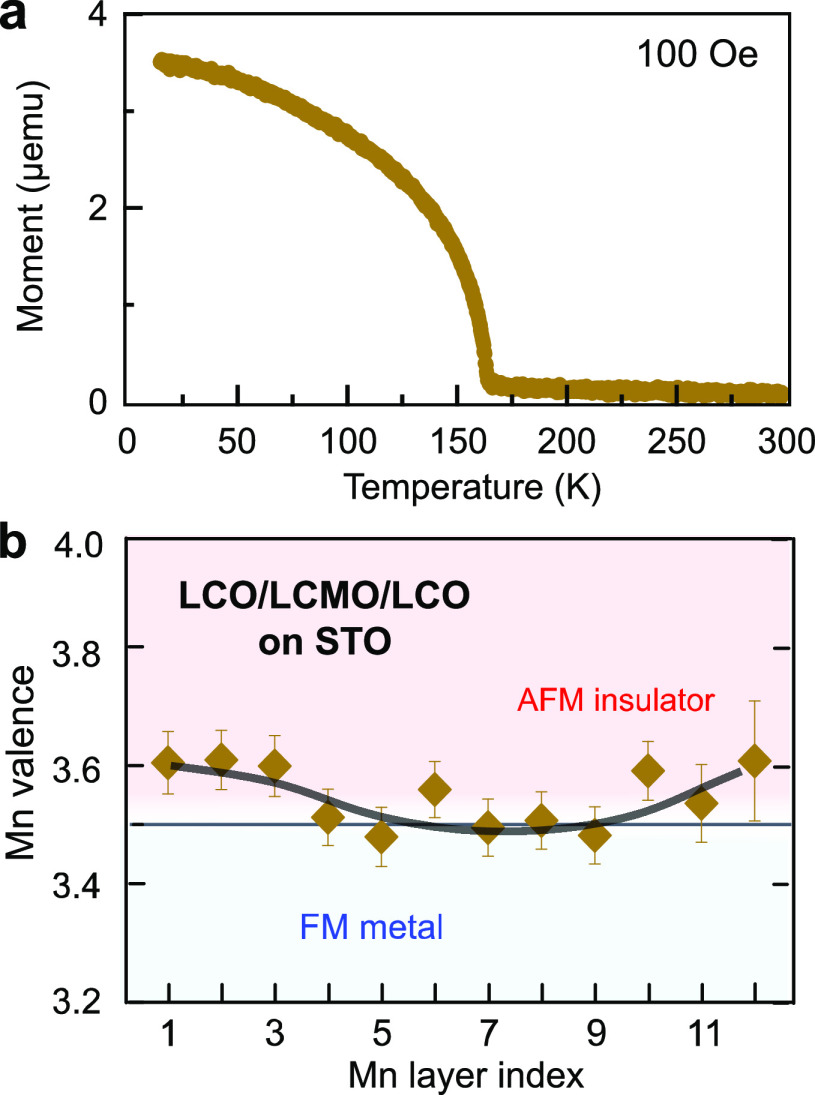
Magnetic property and charge profile in LCO/LCMO/LCO trilayer. (a) Temperature-dependent magnetization curve for the LCO/LCMO/LCO trilayer on STO. The curve was measured after field cooling the samples in a 100 Oe in-plane field. (b) Map of local Mn valence and corresponding local magnetic phase relative to bulk La_0.5_Ca_0.5_MnO_3_ within the LCMO layer. Thick solid lines on charge profiles are guides to the eye.

To confirm the tunability of the charge delocalization and the magnetic phase in trilayers, we also investigated a structure consisting of a 10-u.c.-thick La_0.5_Ca_0.5_MnO_3_ (LCMO) sandwiched by LCO grown on STO, since the size of the A-site ions in the manganites also influences the stability of the structural phase and may induce chemical pressure. LCMO (*a*_0_ = 3.83 Å) is tensile-strained on STO with a lattice mismatch of δ = −1.96%. Here, the interfacial structure follows a similar sequence compared to the LCO/LSMO/LCO trilayer, with the only difference that less deficiency of the dopant concentration is observed at the bottom interface (Figures S10, S11). If the A-site intermixing is responsible for the charge redistribution, we expect to observe some differences in the Mn valence at the interface between two trilayers. Nevertheless, an increased Mn valence close to 3.6+ near the bottom interface occurs in both films (Figure 5b), verifying that changes in the Mn valence are related to the B-site rather than the A-site sublattice ions. Moreover, we found a weaker ferromagnetism in the LCO/LCMO/LCO trilayer (Figure 5a), compared with LCO/LSMO/LCO on STO. Owing to a stronger chemical pressure induced by the smaller ionic radius of Ca, this allows the lattice to extend the tetragonal phase toward a lower *c*/*a* ratio range (Table S1), which decreases the extent of the charge redistribution. As a consequence, a further weakening of magnetism in manganite layers is observed here.

### Effect of Strain on Charge Distribution and Magnetism

**Figure 6:**
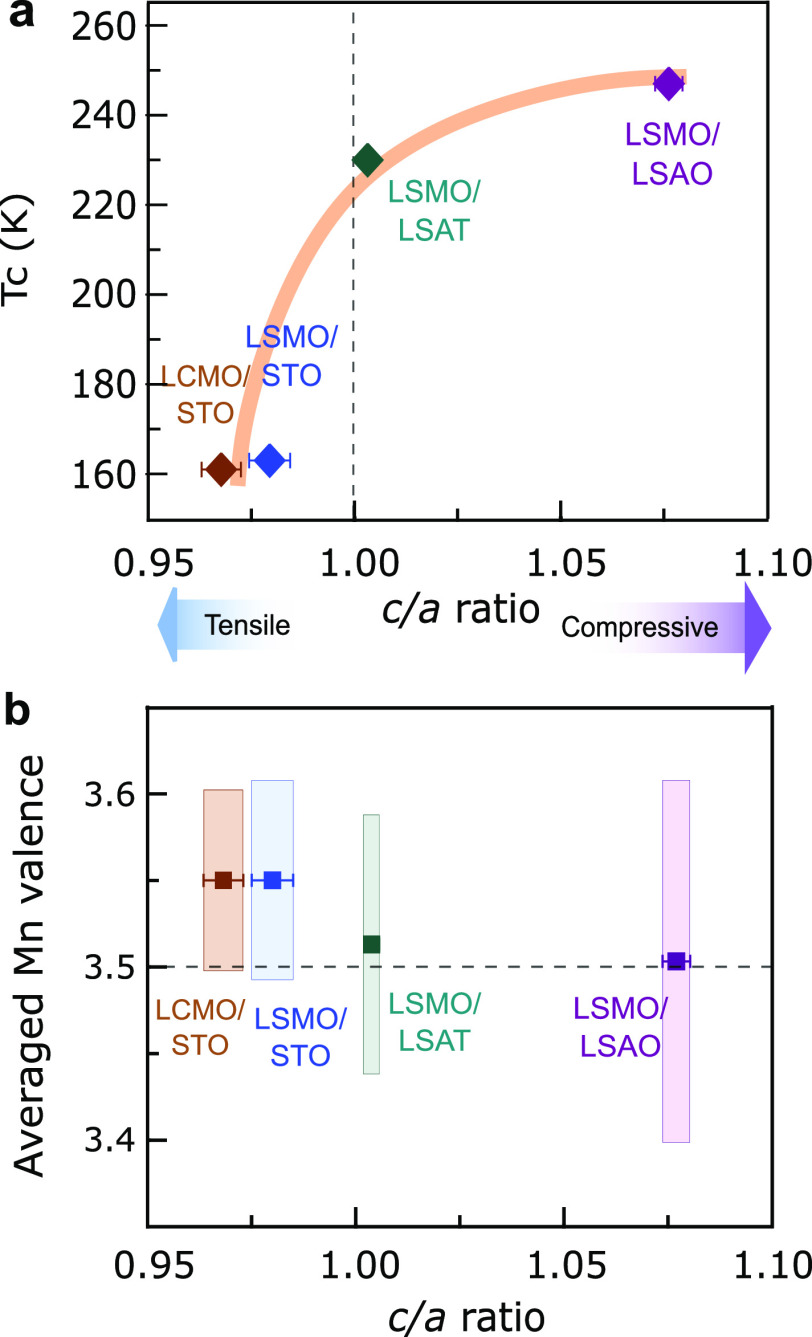
Role of strain on magnetism and charge distribution. (a) Curie temperature (*T*_C_) versus the *c*/*a* ratio within LSMO layers for the four samples. The *c*/*a* ratio is calculated using Mn–Mn interatomic distances in manganite layers (Figure S3). The error bars correspond to the standard deviation of the average of 12 and 20 Mn layers along the out-of-plane (*c*) and in-plane (*a*) directions, respectively. (b) Averaged Mn valence of manganite layers from Figures 4b and 5b versus the *c*/*a* ratio. The length of bars is the standard deviation of the mean, showing the magnitude of Mn valence variation. The dash line is the nominal 3.5+ Mn valence for stoichiometric LSMO.

To elucidate the role of the structure in the charge redistribution and magnetism within the individual LSMO layers, we compare the *c*/*a* ratio variation as a function of the Mn valence and Curie temperature. First, the Curie temperature increases as the averaged *c*/*a* ratio of LSMO layer is increased (Figure 6a), which is at strong variance with the previously reported suppressed magnetism on single-layered LSMO films by substrate-induced strain.^22,23^ This suggests that the mechanism should involve other aspects of the interface besides the Jahn–Teller effect. Second, we find that the average Mn valence decreases with increasing averaged *c*/*a* ratio (Figure 6b), indicating a correlation between the amount of transferred electrons from manganites and the lattice strain. A change in the magnitude of the charge redistribution is also observed from the standard deviations of the means. The trilayer on STO shows a Mn valence of 3.55+ averaged over the whole manganite layer, higher than the expected nominal 3.5+ Mn valence. This is consistent with the scenario of the charge transferred from the manganite to the cuprate^8,9^ and suggests an intrinsic mechanism due to the interfacial electronic reconstruction. In contrast, under compressive strain, the significantly large spatial variation of the Mn valence in LSMO layer with an averaged Mn valence of ∼3.5+ suggests less transferred charge from manganite and a driving force involving more extrinsic effects, e.g., chemical intermixing at the interface.

## Conclusions

Combining strain and interface effects allows us to establish the link between the structural and electronic reconfiguration at the cuprate-manganite interfaces. Near the interface, the observed hole-accumulation-induced AF exchange coupling can be dominated by a combination of charge transfer (due to band mismatch) and the Cu/Mn intermixing based on the similar length scale with the increased Mn valence. Such charge redistribution in the LSMO layer can be attributed to the electrostatic interaction. It is possible that a substitution of Cu^2+^ on the Mn^3.5+^ site at the interface as a hole donor attracts the holes (Mn^4+^) toward the negatively charged interface. Hence, a lowered Mn valence in the central LSMO layer is observed. Another possible scenario, which could realize the observed redistribution, is that due to the size mismatch between Cu^2+^, Mn^3+^ (∼0.7 Å), and Mn^4+^ (∼0.5 Å),^40^ the diffusion of larger Cu^2+^ at the interface causes Mn^3+^ moving into the central layer to relax the elastic strain energy.^41^ Meanwhile, the lattice strain plays an important role in affecting the magnitude of the charge redistribution within manganite layers. The compressive tetragonal distortion produces a lowering of the 3*z*^2^–*r*^2^ orbitals, leading to a stronger delocalization of electrons in the out-of-plane direction.^19^ Therefore, the effect of strain together with the Cu/Mn substitution may result in a larger variation of the Mn valence for the trilayer on LSAO, and an electron enrichment away from the interface, which is presumably responsible for its enhanced FM and metallic behavior. On the other hand, the tensile strain favors the occupation of the *x*^2^–*y*^2^ orbitals. This leads to confinement of electrons in the in-plane direction and a reduced charge redistribution in the LSMO layer.

In summary, we visualize the strain-tuned charge redistribution by mapping local Mn valence variations in manganite layers. These results emphasize the importance of the interface effect, which here leads to a prominent charge redistribution away from the interface and alters its magnetic and electronic structure drastically. Further, the lattice strain together with the Cu/Mn substitution can modify the charge delocalization at the interface. This finding may provide opportunities to tune the charge transfer at cuprate/manganite interfaces. More broadly, our approach of engineering the spatial extent of the charge redistribution can be applied to achieve a more precise property control at the atomic scale for oxide electronics and related devices.

## Methods

### Thin Film Fabrication

LCO/LSMO/LCO trilayers were grown by using an ozone-assisted atomic-layer-by-layer oxide MBE system. The deposition conditions used for synthesizing the samples were a temperature of ∼620 °C (pyrometer reading) and a pressure of ∼1 × 10^–5^ Torr (of mixed ozone and molecular and atomic oxygen). Each individual growth step was monitored by using *in situ* reflection high-energy electron diffraction (RHEED). Representative RHEED patterns taken from individual LCO and LSMO layers of the trilayer sample grown on LSAT substrate are presented in Figure S1 as an example. The structural quality of the films was confirmed *ex situ* by high-resolution X-ray diffraction (see Figure S2).

### Electron Microscopy and Spectroscopy

The TEM sample preparation includes mechanical grinding (down to ∼10 μm), tripod wedge polishing (with an angle of ∼1.5°), and double-sided argon-ion milling. For argon-ion thinning, a precision ion polishing system II (PIPS, Model 695) was used at low temperature. Immediately before the experiment, samples were treated in a Fischione plasma cleaner in a 75% argon–25% oxygen mixture. For STEM analysis, a probe-aberration-corrected JEOL JEM-ARM200F STEM equipped with a cold field-emission electron source, a probe Cs-corrector (DCOR, CEOS GmbH), a Gatan GIF Quantum ERS spectrometer, and a Gatan K2 direct electron detector was used at 200 kV. STEM imaging and EELS analyses were performed at probe semiconvergence angles of 20 and 28 mrad, resulting in probe sizes of 0.8 and 1.0 Å, respectively. The collection angle range for HAADF imaging was 110–270 mrad. A collection semiangle of 111 mrad was used for EELS investigations. A 0.5 eV/ch dispersion with an effective energy resolution of ∼1 eV was used for overall chemical profiling of the films, and 0.1 eV/ch dispersion with an effective energy resolution of ∼0.5 eV was chosen particularly for the Mn *L*_2,3_ white lines to quantify the Mn *L*_3_/*L*_2_ intensity ratio. Further details of the data processing and the corresponding Figure S12 are given in Supporting Information.

### Electronic and Magnetic Properties

We used SQUID magnetometry to measure the magnetic properties. The magnetization curves were measured using a Magnetic Property Measurement System (MPMS, Quantum Design Co.) in the Vibrating Sample Magnetometer (VSM) mode. Electrical measurements were done in a Van der Pauw (four-point-probe) configuration using alternative DC currents of ±20 μA. The values of resistivity at room temperature (300 K) are 0.14, 0.2, and 1.57 mΩ cm in the trilayer on LSAO, LSAT, and STO, respectively.

## Supporting Information Available

Representative RHEED images acquired during the growth, XRD θ–2θ scans, Mn–Mn interatomic distances for all trilayers, table with measured lattice parameters and *c*/*a* ratios, elemental maps in grayscale, elemental distribution line profiles, wide-range magnetic hysteresis loops of the film on LSAT, magnetic hysteresis loops of the film on STO and LSAO, temperature-dependent magnetization curves, elemental distribution for the LCO/LCMO/LCO trilayer on STO, elemental distribution at LCO/LSMO and LCO/LCMO bottom interfaces, Mn *L*_3_/*L*_2_ ratio analysis, and supplementary description for data processing (PDF)

## References

[ref1] Tokura Y. Orbital Physics in Transition-Metal Oxides. Science 2000, 288, 462–468. 10.1126/science.288.5465.462. 10775098

[ref2] Hwang H. Y.; Iwasa Y.; Kawasaki M.; Keimer B.; Nagaosa N.; Tokura Y. Emergent Phenomena at Oxide Interfaces. Nat. Mater. 2012, 11, 103–113. 10.1038/nmat3223. 22270825

[ref3] Ramesh R.; Schlom D. G. Creating Emergent Phenomena in Oxide Superlattices. Nat. Rev. Mater. 2019, 4, 257–268. 10.1038/s41578-019-0095-2.

[ref4] Sefrioui Z.; Arias D.; Pena V.; Villegas J. E.; Varela M.; Prieto P.; Leon C.; Martinez J. L.; Santamaria J. Ferromangetic/Superconducting Proximity Effect in La_0.7_Sr_0.3_MnO_3_/YBa_2_Cu_3_O_7–*x*_ Superlattices. Phys. Rev. B: Condens. Matter Mater. Phys. 2003, 67, 214511 10.1103/PhysRevB.67.214511.

[ref5] Chakhalian J.; Freeland J. W.; Srajer G.; Strempfer J.; Khaliullin G.; Cezar J. C.; Charlton T.; Dalgliesh R.; Bernhard C.; Cristiani G.; Habermeier H.-U.; Keimer B. Magnetism at the Interface between Ferromagnetic and Superconducting Oxides. Nat. Phys. 2006, 2, 244–248. 10.1038/nphys272.

[ref6] Hoppler J.; Stahn J.; Niedermayer C.; Malik V. K.; Bouyanfif H.; Drew A. J.; Rössle M.; Buzdin A.; Cristiani G.; Habermeier H.-U.; Keimer B.; Bernhard C. Giant Superconductivity-Induced Modulation of the Ferromagnetic Magnetization in a Cuprate–Manganite Superlattice. Nat. Mater. 2009, 8, 315–319. 10.1038/nmat2383. 19219030

[ref7] Driza N.; Blanco-Canosa S.; Bakr M.; Soltan S.; Khalid M.; Mustafa L.; Kawashima K.; Christiani G.; Habermeier H.-U.; Khaliullin G.; Ulrich C.; Le Tacon M.; Keimer B. Long-Range Transfer of Electron–Phonon Coupling in Oxide Superlattices. Nat. Mater. 2012, 11, 675–681. 10.1038/nmat3378. 22797829

[ref8] Yunoki S.; Moreo A.; Dagotto E.; Okamoto S.; Kancharla S. S.; Fujimori A. Electron Doping of Cuprates via Interfaces with Manganites. Phys. Rev. B: Condens. Matter Mater. Phys. 2007, 76, 064532 10.1103/PhysRevB.76.064532.

[ref9] Chakhalian J.; Freeland J. W.; Habermeier H.-U.; Cristiani G.; Khaliullin G.; van Veenendaal M.; Keimer B. Orbital Reconstruction and Covalent Bonding at an Oxide Interface. Science 2007, 318, 1114–1117. 10.1126/science.1149338. 17932255

[ref10] Visani C.; Tornos J.; Nemes N. M.; Rocci M.; Leon C.; Santamaria J.; te Velthuis S. G. E.; Liu Y.; Hoffmann A.; Freeland J. W.; Garcia-Hernandez M.; Fitzsimmons M. R.; Kirby B. J.; Varela M.; Pennycook S. J. Symmetrical Interfacial Reconstruction and Magnetism in La_0.7_Sr_0.3_MnO_3_/YBa_2_Cu_3_O_7–*x*_/La_0.7_Sr_0.3_MnO_3_ Heterostructures. Phys. Rev. B: Condens. Matter Mater. Phys. 2011, 84, 060405 10.1103/PhysRevB.84.060405.

[ref11] Seo A.; Boris A. V.; Cristiani G.; Habermeier H.-U.; Keimer B. Optical Characteristics of Charge Carrier Transfer across Interfaces between YBa_2_Cu_3_O_6+δ_ and La_0.7_Sr_0.3_MnO_3_. Phys. Rev. B: Condens. Matter Mater. Phys. 2019, 99, 064501 10.1103/PhysRevB.99.064501.

[ref12] Chien T. Y.; Kourkoutis L. F.; Chakhalian J.; Gray B.; Kareev M.; Guisinger N. P.; Muller D. A.; Freeland J. W. Visualizing Short-Range Charge Transfer at the Interfaces between Ferromagnetic and Superconducting Oxides. Nat. Commun. 2013, 4, 2336 10.1038/ncomms3336. 23939385

[ref13] Salafranca J.; Rincón J.; Tornos J.; León C.; Santamaria J.; Dagotto E.; Pennycook S. J.; Varela M. Competition between Covalent Bonding and Charge Transfer at Complex-Oxide Interfaces. Phys. Rev. Lett. 2014, 112, 196802 10.1103/PhysRevLett.112.196802. 24877959

[ref14] Hoffmann A.; te Velthuis S. G. E.; Sefrioui Z.; Santamaría J.; Fitzsimmons M. R.; Park S.; Varela M. Suppressed Magnetization in La_0.7_Sr_0.3_MnO_3_/YBa_2_Cu_3_O_7–*x*_. Phys. Rev. B: Condens. Matter Mater. Phys. 2005, 72, 140407 10.1103/PhysRevB.72.140407.

[ref15] Tebano A.; Aruta C.; Sanna S.; Medaglia P. G.; Balestrino G.; Sidorenko A. A.; De Renzi R.; Ghiringhelli G.; Braicovich L.; Bisogni V.; Brookes N. B. Evidence of Orbital Reconstruction at Interfaces in Ultrathin La_0.67_Sr_0.33_MnO_3_ Films. Phys. Rev. Lett. 2008, 100, 137401 10.1103/PhysRevLett.100.137401. 18517994

[ref16] Huijben M.; Martin L. W.; Chu Y.-H.; Holcomb M. B.; Yu P.; Rijnders G.; Blank D. H. A.; Ramesh R. Critical Thickness and Orbital Ordering in Ultrathin La_0.7_Sr_0.3_MnO_3_ Films. Phys. Rev. B: Condens. Matter Mater. Phys. 2008, 78, 094413 10.1103/PhysRevB.78.094413.

[ref17] Dong S.; Yunoki S.; Zhang X.; Şen C.; Liu J.-M.; Dagotto E. Highly Anisotropic Resistivities in the Double-Exchange Model for Strained Manganites. Phys. Rev. B: Condens. Matter Mater. Phys. 2010, 82, 035118 10.1103/PhysRevB.82.035118.

[ref18] Nanda B. R. K.; Satpathy S. Effects of Strain on Orbital Ordering and Magnetism at Perovskite Oxide Interfaces: LaMnO_3_/SrMnO_3_. Phys. Rev. B: Condens. Matter Mater. Phys. 2008, 78, 054427 10.1103/PhysRevB.78.054427.

[ref19] Millis A. J.; Shraiman B. I.; Mueller R. Dynamic Jahn-Teller Effect and Colossal Magnetoresistence in La_1–*x*_Sr_*x*_MnO_3_. Phys. Rev. Lett. 1996, 77, 175–178. 10.1103/PhysRevLett.77.175. 10061800

[ref20] Konishi Y.; Fang Z.; Izumi M.; Manako T.; Kasai M.; Kuwahara H.; Kawasaki M.; Terakura K.; Tokura Y. Orbital-State-Mediated Phase-Control of Manganites. J. Phys. Soc. Jpn. 1999, 68, 3790–3793. 10.1143/JPSJ.68.3790.

[ref21] Tsui F.; Smoak M. C.; Nath T. K.; Eom C. B. Strain-Dependent Magnetic Phase Diagram of Epitaxial La_0.67_Sr_0.33_MnO_3_ Thin Films. Appl. Phys. Lett. 2000, 76, 2421–2423. 10.1063/1.126363.

[ref22] Pesquera D.; Barla A.; Wojcik M.; Jedryka E.; Bondino F.; Magnano E.; Nappini S.; Gutiérrez D.; Radaelli G.; Herranz G.; Sánchez F.; Fontcuberta J. Strain-Driven Orbital and Magnetic Orders and Phase Separation in Epitaxial Half-Doped Manganite Films for Tunneling Devices. Phys. Rev. Appl. 2016, 6, 034004 10.1103/PhysRevApplied.6.034004.

[ref23] Gutiérrez D.; Radaelli G.; Sánchez F.; Bertacco R.; Fontcuberta J. Bandwidth-Limited Control of Orbital and Magnetic Orders in Half-Doped Manganites by Epitaxial Strain. Phys. Rev. B: Condens. Matter Mater. Phys. 2014, 89, 075107 10.1103/PhysRevB.89.075107.

[ref24] Suyolcu Y. E.; Christiani G.; van Aken P. A.; Logvenov G. Design of Complex Oxide Interfaces by Oxide Molecular Beam Epitaxy. J. Supercond. Novel Magn. 2020, 33, 107–120. 10.1007/s10948-019-05285-4.

[ref25] Fujishiro H.; Fukase T.; Ikebe M. Charge Ordering and Sound Velocity Anomaly in La_1–*x*_Sr _*x*_MnO_3_ (*x* ≥ 0.5). J. Phys. Soc. Jpn. 1998, 67, 2582–2585. 10.1143/JPSJ.67.2582.

[ref26] Fujishiro H.; Ikebe M.; Konno Y. Phase Transition to Antiferromagnetic State in La_1–*x*_Sr _*x*_MnO_3_ (*x* ≥ 0.5). J. Phys. Soc. Jpn. 1998, 67, 1799–1800. 10.1143/JPSJ.67.1799.

[ref27] Sun J. Z.; Abraham D. W.; Rao R. A.; Eom C. B. Thickness-Dependent Magnetotransport in Ultrathin Manganite Films. Appl. Phys. Lett. 1999, 74, 3017–3019. 10.1063/1.124050.

[ref28] Kim G.; Khaydukov Y.; Bluschke M.; Suyolcu Y. E.; Christiani G.; Son K.; Dietl C.; Keller T.; Weschke E.; van Aken P. A.; Logvenov G.; Keimer B. Tunable Perpendicular Exchange Bias in Oxide Heterostructures. Phys. Rev. Mater. 2019, 3, 084420 10.1103/PhysRevMaterials.3.084420.

[ref29] Suyolcu Y. E.; Sun J.; Goodge B. H.; Park J.; Schubert J.; Kourkoutis L. F.; Schlom D. G. a-Axis YBa_2_Cu_3_O_7–*x*_/PrBa_2_Cu_3_O_7–*x*_/YBa_2_Cu_3_O_7–*x*_ Trilayers with Subnanometer Rms Roughness. APL Mater. 2021, 9, 021117 10.1063/5.0034648.

[ref30] Baiutti F.; Gregori G.; Wang Y.; Suyolcu Y. E.; Cristiani G.; van Aken P. A.; Maier J.; Logvenov G. Cationic Redistribution at Epitaxial Interfaces in Superconducting Two-Dimensionally Doped Lanthanum Cuprate Films. ACS Appl. Mater. Interfaces 2016, 8, 27368–27375. 10.1021/acsami.6b09739. 27648928

[ref31] Dagotto E.; Hotta T.; Moreo A. Colossal Magnetoresistant Materials: The Key Role of Phase Separation. Phys. Rep. 2001, 344, 1–153. 10.1016/S0370-1573(00)00121-6.

[ref32] Cui B.; Song C.; Wang G. Y.; Mao H. J.; Zeng F.; Pan F. Strain Engineering Induced Interfacial Self-Assembly and Intrinsic Exchange Bias in a Manganite Perovskite Film. Sci. Rep. 2013, 3, 2542 10.1038/srep02542. 23985971 PMC3756339

[ref33] Scholten G.; Usadel K. D.; Nowak U. Coercivity and Exchange Bias of Ferromagnetic/Antiferromagnetic Multilayers. Phys. Rev. B: Condens. Matter Mater. Phys. 2005, 71, 064413 10.1103/PhysRevB.71.064413.

[ref34] De Luca G. M.; Ghiringhelli G.; Perroni C. A.; Cataudella V.; Chiarella F.; Cantoni C.; Lupini A. R.; Brookes N. B.; Huijben M.; Koster G.; Rijnders G.; Salluzzo M. Ubiquitous Long-Range Antiferromagnetic Coupling across the Interface between Superconducting and Ferromagnetic Oxides. Nat. Commun. 2014, 5, 5626 10.1038/ncomms6626. 25418631

[ref35] Ding J.; Cossu F.; Lebedev O. I.; Zhang Y.; Zhang Z.; Schwingenschlögl U.; Wu T. Manganite/Cuprate Superlattice as Artificial Reentrant Spin Glass. Adv. Mater. Interfaces 2016, 3, 1500676 10.1002/admi.201500676.

[ref36] Goodenough J. B. Theory of the Role of Covalence in the Perovskite-Type Manganites [La, M(II)]MnO_3_. Phys. Rev. 1955, 100, 564–573. 10.1103/PhysRev.100.564.

[ref37] Varela M.; Oxley M. P.; Luo W.; Tao J.; Watanabe M.; Lupini A. R.; Pantelides S. T.; Pennycook S. J. Atomic-Resolution Imaging of Oxidation States in Manganites. Phys. Rev. B: Condens. Matter Mater. Phys. 2009, 79, 085117 10.1103/PhysRevB.79.085117.

[ref38] Biswas A.; Rajeswari M.; Srivastava R. C.; Venkatesan T.; Greene R. L.; Lu Q.; de Lozanne A. L.; Millis A. J. Strain-Driven Charge-Ordered State in La_0.67_Ca_0.33_MnO_3_. Phys. Rev. B: Condens. Matter Mater. Phys. 2001, 63, 184424 10.1103/PhysRevB.63.184424.

[ref39] Baena A.; Brey L.; Calderón M. J. Effect of Strain on the Orbital and Magnetic Ordering of Manganite Thin Films and Their Interface with an Insulator. Phys. Rev. B: Condens. Matter Mater. Phys. 2011, 83, 064424 10.1103/PhysRevB.83.064424.

[ref40] Shannon R. D. Revised Effective Ionic Radii and Systematic Studies of Interatomic Distances in Halides and Chalcogenides. Acta Crystallogr., Sect. A: Cryst. Phys., Diffr., Theor. Gen. Crystallogr. 1976, 32, 751–767. 10.1107/S0567739476001551.

[ref41] Lee W.; Han J. W.; Chen Y.; Cai Z.; Yildiz B. Cation Size Mismatch and Charge Interactions Drive Dopant Segregation at the Surfaces of Manganite Perovskites. J. Am. Chem. Soc. 2013, 135, 7909–7925. 10.1021/ja3125349. 23642000

