# Supplementary material for: Atomic-Scale Tuning of the Charge Distribution by Strain Engineering in Oxide Heterostructures
Source: ACS Nano. 2021 Sep 30;15(10):16228–35. doi: 10.1021/acsnano.1c05220 (PMC8552499; doi:10.1021/acsnano.1c05220)
Supplement: Supplementary file 1 — nn1c05220_si_001.pdf [file nn1c05220_si_001.pdf]

Supporting Information
Atomic-Scale Tuning of the Charge Distribution by Strain Engineering in Oxide Heterostructures

Yu-Mi Wu,[*,†] Y. Eren Suyolcu,[*,†,‡] Gideok Kim,[†] Georg Christiani,[†] Yi Wang,[†] Bernhard Keimer,[†] Gennady Logvenov,[†] and Peter A. van Aken[†]

†*Max-Planck-Institut für Festkörperforschung, Heisenbergstrasse 1, 70569 Stuttgart, Germany*

‡*Department of Materials Science and Engineering, Cornell University, Ithaca, New York 14853, USA*

E-mail: yu-mi.wu@fkf.mpg.de; eren.suyolcu@cornell.edu

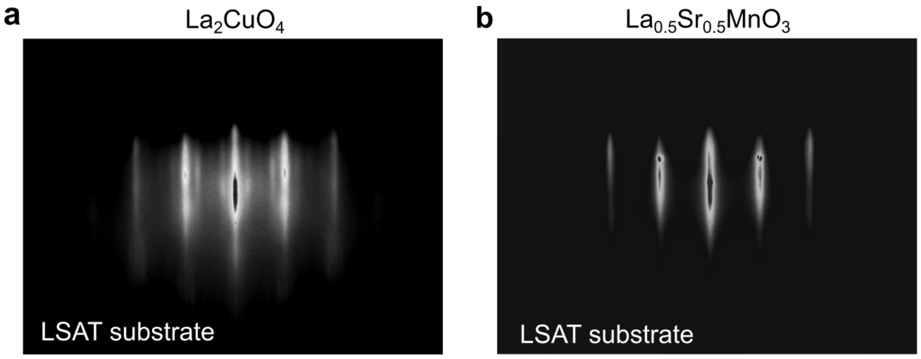

Figure S1: Representative RHEED images acquired during the growth of (a) La_2CuO_4 (LCO) and (b) $La_{0.5}Sr_{0.5}MnO_3$ (LSMO) layers on LSAT (001) substrate.

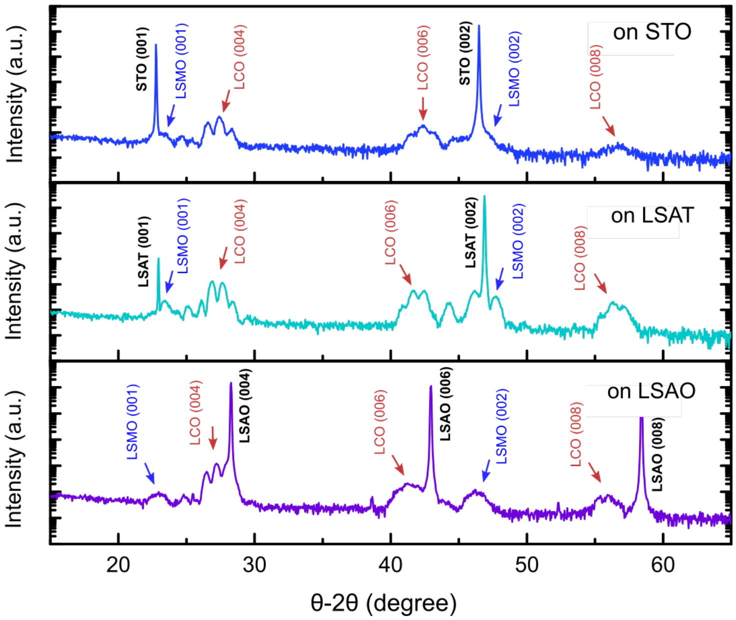

Figure S2: XRD θ-2θ scans for LCO/LSMO/LCO films on STO, LSAT and LSAO substrates, respectively. The presence of thickness fringes around the film peaks indicates the good structural quality.

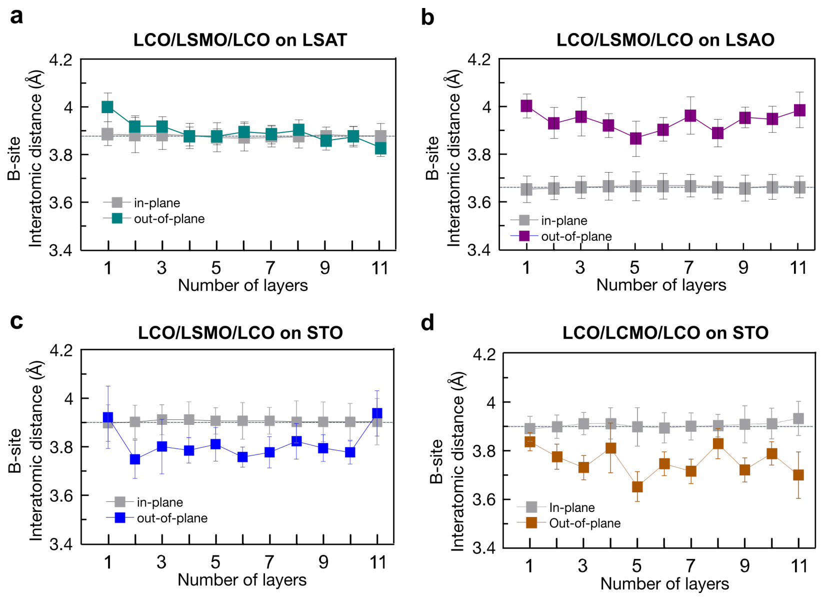

Figure S3: Comparison of Mn-Mn interatomic distances along the in-plane and out-of-plane directions for all trilayers. (a-c) LCO/LSMO/LCO on LSAT, LSAO, and STO, respectively. (d) LCO/LCMO/LCO on STO. The error bars are determined from the standard deviation of the atomic positions of 20 unit cells along the in-plane direction. The slight increase of out-of-plane interatomic distances could be caused by cation intermixing near interfaces, indicative of a suppressed octahedral distortion. Due to the higher Mn valence and the suppressed distortion at the interface, the lack of carriers and the reduced in-plane electron hopping by increasing the Mn-O-Mn bond length lead to a reduction of the conductivity and FM double-exchange interaction.[1]

Table S1: Measured lattice parameters and c/a ratios of LSMO layers based on the Mn-Mn interatomic distances averaged along the in-plane (a) and out-of-plane (c) directions.

	LSMO on LSAO	LSMO on LSAT	LSMO on STO	LCMO on STO
a (Å)	3.66	3.88	3.9	3.9
c (Å)	3.94	3.89	3.82	3.76
c/a ratio	1.077	1.0038	0.98	0.96

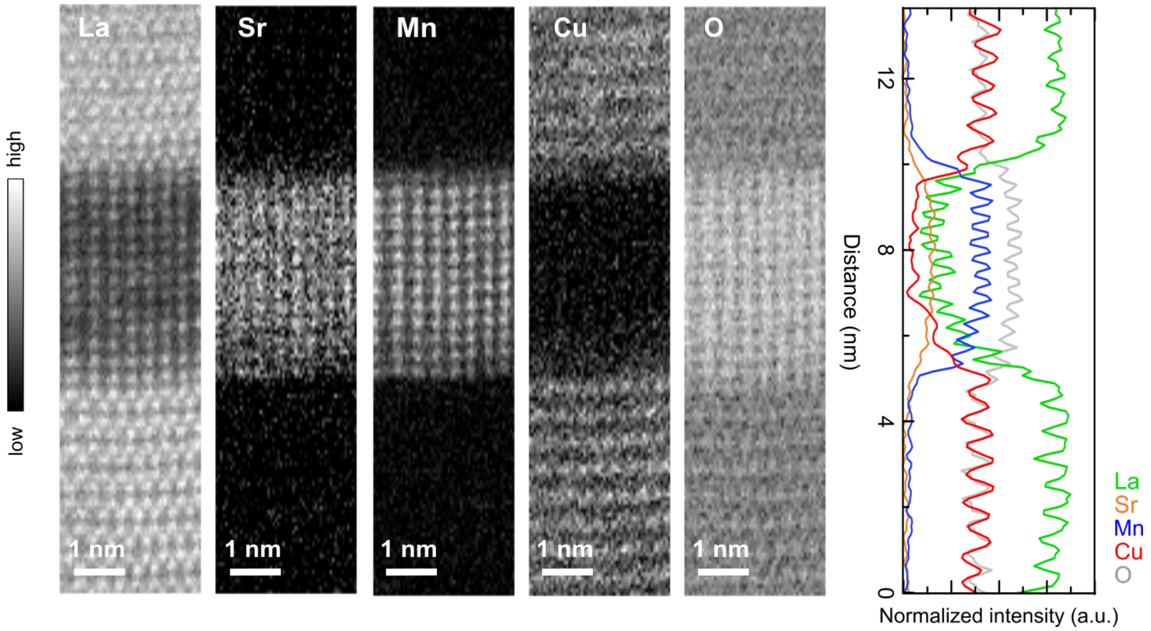

Figure S4: Elemental concentration maps of La, Sr, Mn, Cu and O for LCO/LSMO/LCO trilayers on LSAT in grayscale. The right panel is the horizontally integrated intensity profiles normalized by the whole compositions obtained from the maps.

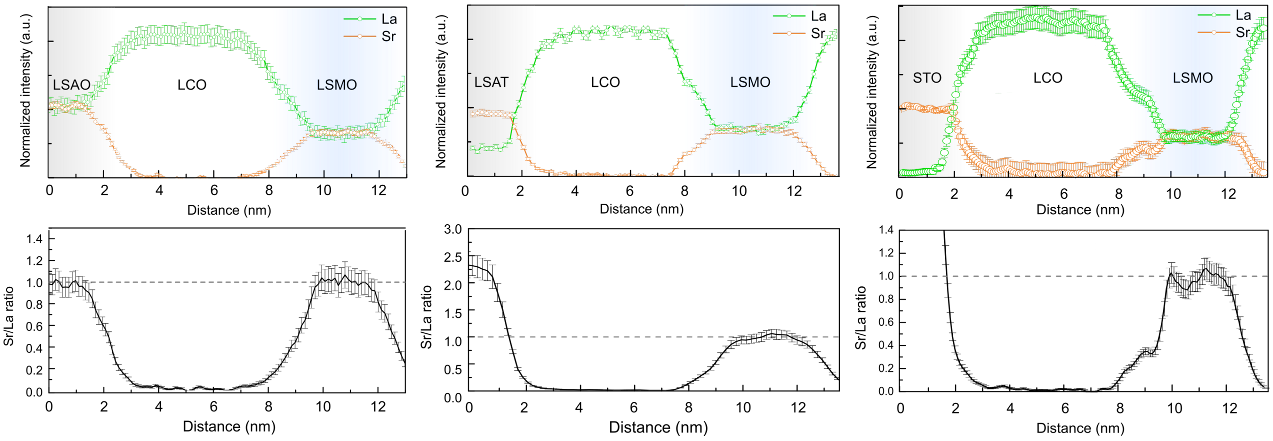

Figure S5: Intensity profiles of La in green and Sr in orange (upper panel) and Sr/La ratio (lower panel) as a function of distance from the substrate for LCO/LSMO/LCO trilayers on STO, LSAT and LSAO, respectively. All three trilayers show the averaged ratio of ~ 1, confirming the Sr/La stoichiometry of LSMO layers.

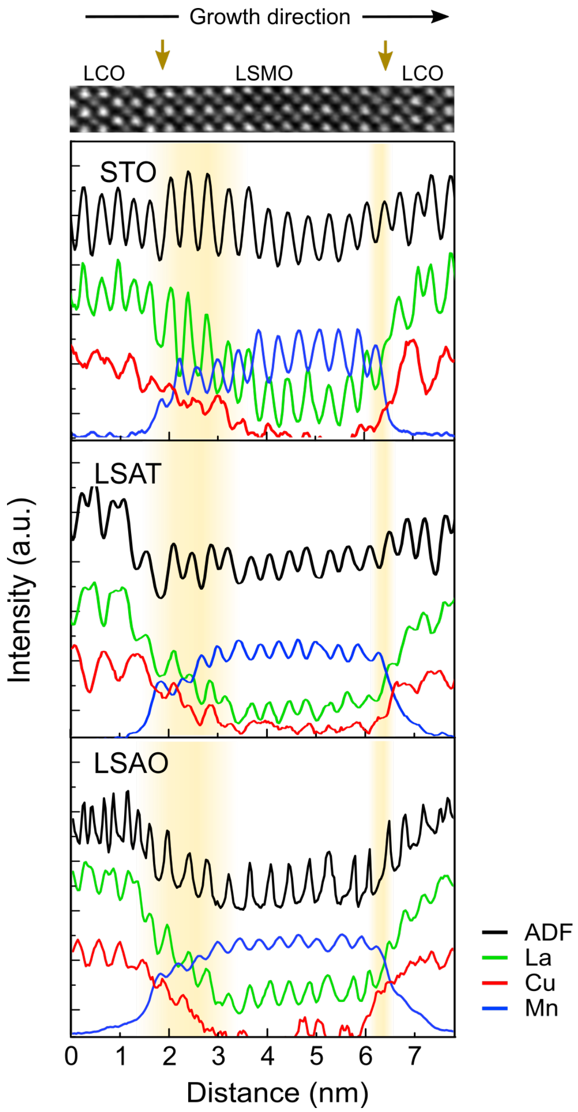

Figure S6: Elemental distribution line profiles of ADF (black), La (green), Mn (blue) and Cu (red) for LCO/LSMO/LCO trilayers on STO (top image), LSAT and LSAO, respectively. The representative STEM-HAADF image of the trilayer on STO is given as a guide for the eyes. The yellow arrows and the shaded regions indicate the two interfaces and the width of cation intermixing at the interfaces. All three trilayers exhibit two additional Mn layers and broader cation intermixing at the bottom interface over ∼1.5 nm compared to the top interface (∼0.5 nm).

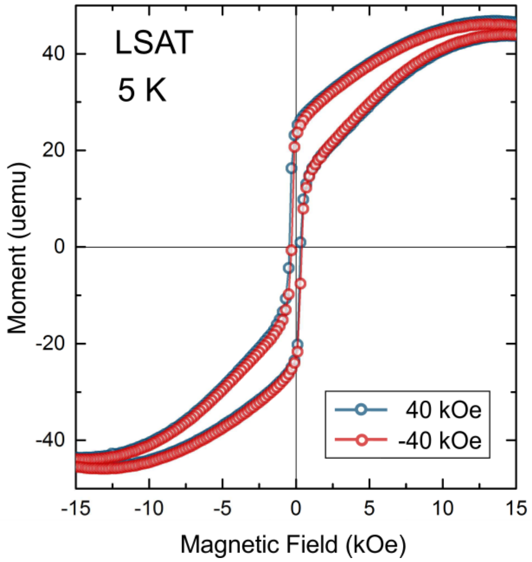

Figure S7: Wide-range magnetic hysteresis loops of the film on LSAT measured at 5K. The measurements were performed after field cooling in a ± 40 kOe applied in-plane magnetic field. The signal from the substrate was subtracted after the measurements.

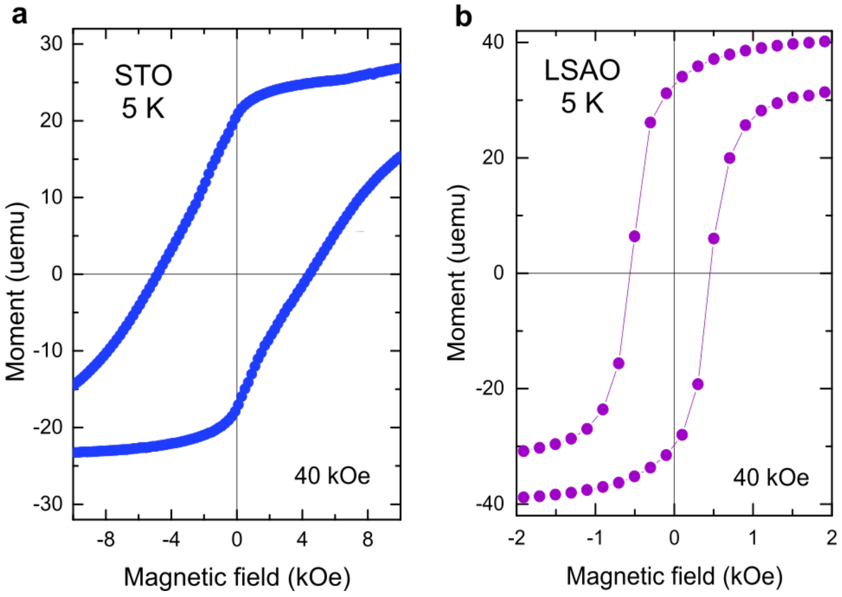

Figure S8: Magnetic hysteresis loops measured at 5K showing exchange bias fields of 195 Oe and 50 Oe in the film on STO (a) and LSAO (b), respectively The measurements were performed after field cooling in a 40 kOe applied in-plane magnetic field. The signal from the substrate was subtracted after the measurements.

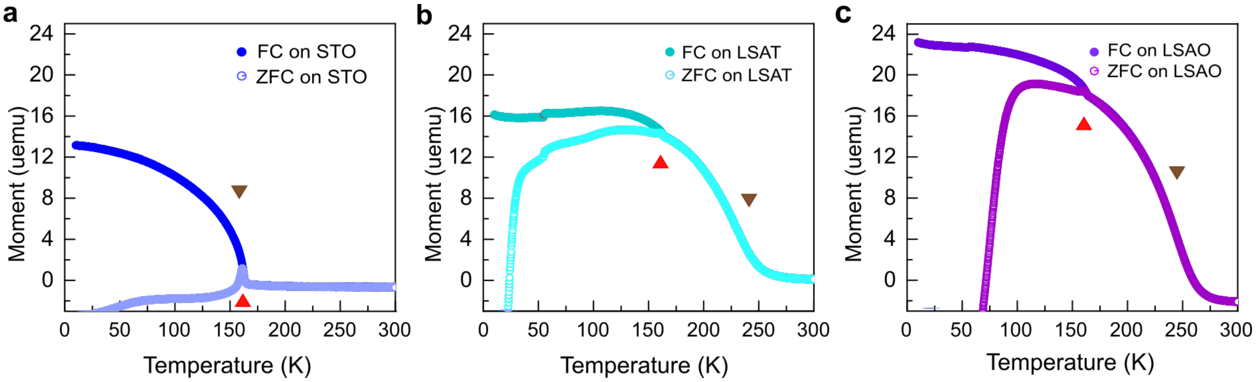

Figure S9: Temperature-dependent magnetization curves of the films on STO (a), LSAT (b) and LSAO (c), respectively. The data were taken in field-cooled (FC) and zero-field-cooled (ZFC) modes with a 100 Oe field applied parallel to in-plane. The brown and red triangle symbols indicate the onset of the ferromagnetic transition and the splitting of ZFC and FC curves, respectively. The ZFC curves all largely bifurcate from the FC curves at $\sim$ 160 K, as an indication of the spin-glass state due to the magnetic frustration.

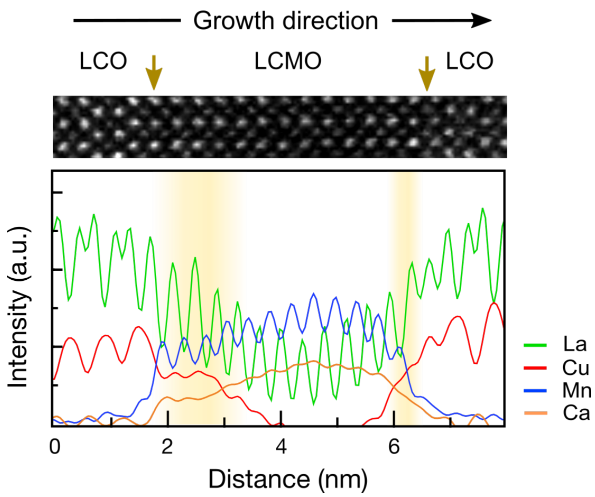

Figure S10: Elemental distribution line profiles of La (green), Mn (blue), Cu (red) and Ca (orange) for the LCO/LCMO/LCO trilayer on STO. The representative simultaneously-recorded STEM-ADF image is given as a guide for the eyes. The yellow arrows and the shaded regions indicate the two interfaces and the width of cation intermixing at the interfaces. The LCO/LCMO/LCO film also shows 2 additional Mn layers and an asymmetry of interface stacking sequences as well as cation intermixing, as observed in LCO/LSMO/LCO trilayers.

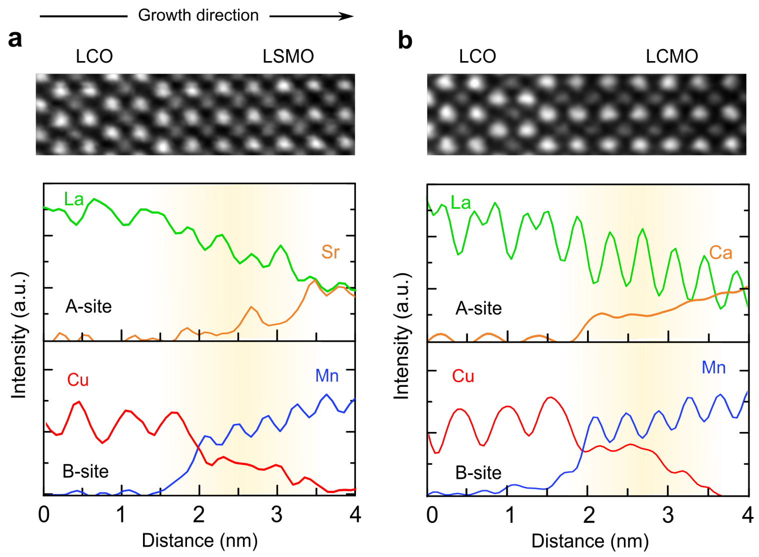

Figure S11: Comparison of elemental distribution at LCO/LSMO and LCO/LCMO bottom interfaces with La in green, Mn in blue, Cu in red and Ca, Sr in orange for LCO/LSMO/LCO (a) and LCO/LCMO/LCO (b). The yellow shaded areas indicate the cation intermixing region. Both films show a similar width of the Cu/Mn intermixing over ~1.5 nm, while the LCO/LSMO interface exhibits more Sr deficiency on the A-site sublattice.

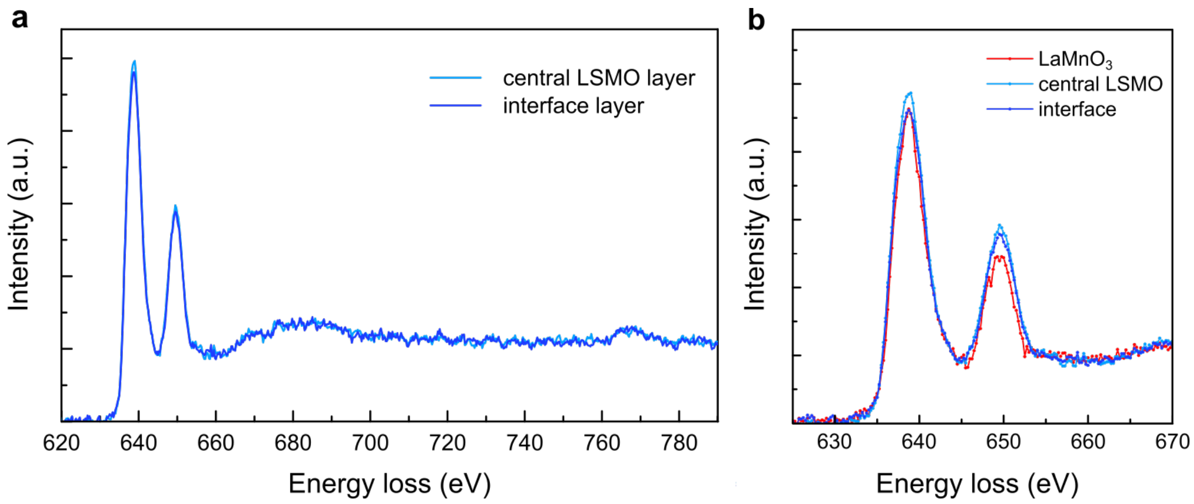

Figure S12: Mn L_3/L_2 intensity ratio analysis from the Mn $L_{3,2}$ spectra. (a) Background-subtracted Mn spectra taken from the central LSMO layer and the interface of the film on LSAT. The spectra are normalized by integration from 655-665 eV, where the scaling window of the step function is used. The continuum intensities shows no difference between two normalized spectra. (b) Zoom-in Mn spectra of the central LSMO layer, interface area of the film on LSAT and a reference spectra for Mn^{3+}. Here the spectra are normalized to the integral intensity in the energy-loss range from 655-665 eV. The L_3/L_2 intensity ratio for Mn^{3+} spectra of $LaMnO_3$ is 2.785, which is consistent with value given in Ref. 37.

Supplementary Description - Data Processing

Each layer-resolved Mn $L_{3,2}$ EELS spectrum of the LSMO layers, shown in Figure 3, is the sum of eight spectra acquired sequentially along the lattice plane parallel to the interface (8 pixels of a spectrum image). Principle components analysis (PCA) was applied to the data to reduce the noise. A power-law background below the Mn $L_{3,2}$ edge was subtracted and the spectra were normalized to the integrated intensity of L_3 edge over approximately 10 eV after the edge onset.

The Mn valence in Figure 3 and 4 are determined from the Mn $L_{3,2}$ spectra of the LSMO layers using the method of ref. 37. The continuum contribution was first removed by scaling a Hartree-Slater cross-section step function as available in Digital Micrograph. A 10 eV-wide window was placed right after the L_2 edge (655-665 eV). The continuum intensity from 665-790 eV shows no or little change between the spectra (Figure S12a). The integrated intensity values over 10 eV under the corrected L_3 and L_2 edges were then used to calculate the L_3/L_2 intensity ratio. For quantitative analysis, a reference spectrum for Mn^{3+} from the bulk of the $LaMnO_3$ film was used to confirm the expected valence state as shown in Figure S12b.

The Mn-Mn interatomic distances in Figure S3 were calculated using frame series of STEM-HAADF images. 10 frames of images were acquired with a short dwell time (2 μs per pixel). The frame series were then aligned and summed to minimize the image distortion and improve signal-to-noise ratio. The distances were calibrated according to the distances measured in the substrates and determined by averaging 20 unit cells along the in-plane direction using the O-O picker software.[2]

Supplementary References

1. Vailionis, A.; Boschker, H.; Siemons, W.; Houwman, E.P.; Blank, D.H.A,; Rijnders, G.; Koster, G. Misfit Strain Accommodation in Epitaxial ABO_3 Perovskites: Lattice Rotations and Lattice Modulations. *Phys. Rev. B* **2011**, *83*, 064101.

2. Wang, Y.; Salzberger, U.; Sigle, W.; Suyolcu, Y. E.; van Aken, P. A. Oxygen Octahedra Picker: A Software Tool to Extract Quantitative Information from STEM Images. *Ultramicroscopy* **2016**, *168*, 46-52.
